# Parastomal hernia repair, trying to optimize the impossible reconstruction

**DOI:** 10.1007/s10029-024-03041-9

**Published:** 2024-04-28

**Authors:** S. M. Maskal, R. C. Ellis, B. T. Miller

**Affiliations:** Department of Surgery, Cleveland Clinic, 2049 E 100th St, Cleveland, OH USA

**Keywords:** Parastomal hernia, Retromuscular Keyhole, Retromuscular Sugarbaker

## Abstract

**Purpose:**

Parastomal hernias are a common and challenging problem with high rates of wound complications and hernia recurrence after repair. We present our approach to optimizing parastomal hernia repair through preoperative preparation, surgical approach, and postoperative management.

**Methods:**

Patients are carefully evaluated and optimized prior to surgery. Our typical surgical approach involves a generous midline laparotomy and retrorectus dissection followed by a posterior component separation with transversus abdominis release. We typically utilize a Sugarbaker technique for retromuscular mesh placement but also use the retromuscular keyhole or cruciate technique if there is insufficient bowel length.

**Results:**

Previously published results from our institution include wound complication rates of up to 16% after open retromuscular parastomal hernia repair. Stoma-specific complications, such as mesh erosion in the bowel, may be attributed to the mesh placement techniques. Hernia recurrence rates range from 11 to 30% up to 2 years postoperatively.

**Conclusion:**

We prefer an open retromuscular approach with a Sugarbaker mesh configuration to treat complex parastomal hernias. However, wound morbidity and repair failure rates remain high, and additional research is needed to optimize surgical outcomes.

## Introduction

Approximately, 450,000 Americans live with an ostomy, of which about 50% will develop a parastomal hernia within the first two years [1, 2]. Parastomal hernia repairs are inherently challenging operations for several reasons, including frequently complex surgical histories, the presence of an unavoidable defect in the abdominal wall needed to accommodate the stoma, the high risk of contamination, and a scarcity of comparative prospective data to guide the surgical approach. This difficulty is reflected in a persistently high recurrence rate, up to 73% in some series [3], and limited guidance from hernia societies based on low-quality evidence [4]. While patients may present with incarcerations prompting emergent repair, elective repair is generally indicated for large parastomal hernias that are symptomatic (i.e., causing obstructions, pain, or poor ostomy appliance fit).

As a tertiary referral center, the parastomal hernias we repair are frequently recurrent (45%), European Hernia Classification IV (49%), and present with a mean defect width of 15.8 cm and a mean defect length of 22.9 cm [5]. Considering the surgical complexity of this population, we typically approach repair through a midline laparotomy with retromuscular mesh placement. Despite the many challenges inherent to parastomal hernia repair, it is critical for hernia surgeons to optimize their reconstructive approach. We aim to describe our typical open retromuscular approach to repairing complex parastomal hernias.

## Methods

### Optimizing the patient

All patients presenting to our institution with parastomal hernias are evaluated with a thorough history and physical examination. Comorbidities are evaluated and optimized as possible. Notably, while weight loss, glycemic control, and smoking cessation are recommended as applicable, we do not typically withhold elective repairs in symptomatic patients based on these risk factors alone. We routinely obtain non-contrasted computed tomography scans to assess abdominal wall and hernia anatomy, concomitant intraabdominal pathology, and evidence of previous repairs. It is important to consider the underlying disease process that led to the creation of a stoma as many of these patients are medically complex. Based on the etiology of the stoma, additional diagnostic studies or consultations may be warranted, for example, to assess candidacy for stoma reversal, evaluate ureteral length and angulation of ileal conduits, and to monitor disease recurrence in the setting of malignancy. For complex patients requiring additional intraabdominal operations, a staged approach to parastomal hernia repair should be considered [6].

### Operative preparation

Patients with planned ostomy relocation undergo preoperative consultation with a certified stoma nurse prior to surgery to identify optimal stoma locations. Of note, stoma location management preference is addressed preoperatively and reflects joint decision-making between the patient and surgeon. Patients are positioned supine with arms out and receive venous thromboembolism prophylaxis, the surgical site is prepped, and preoperative intravenous antibiotics are administered according to SCIP guidelines [7]. Intestinal stomas are infrequently sutured closed at the discretion of the surgeon, but are ubiquitously covered with a sponge and transparent dressing to limit contamination. For ileal conduits, a sterile catheter is placed into the stoma after draping to allow for intraoperative urinary drainage.

### Adhesiolysis and stoma management

A generous midline laparotomy is made, starting at least several centimeters cephalad to any previous scar tissue, which is excised. The native linea alba is identified at the superior aspect of the incision and is then divided progressively caudad as adhesions are sharply lysed free from the undersurface. Once the linea alba is fully divided, a full adhesiolysis of all adhesions to the anterior abdominal wall is performed sharply. Previous mesh is generally excised if present. The parastomal hernia is then reduced and stoma management is considered. If the stoma is being left in situ, which is our preference, the stoma bowel adhesions must be carefully dissected from the abdominal wall aperture. If the stoma is re-sited or re-matured in the same location, then the mucocutaneous junction is divided and the bowel is dissected free from the aperture before dividing the distal end with a linear stapler.

### Component separation

A moist towel is placed over the viscera for protection during the retromuscular dissection, then the hernia defect width and length are measured with a ruler. The retrorectus plane is entered lateral to the linea alba, with visualization of the rectus muscle as confirmation of the correct location, and the posterior rectus sheath is incised along the length of the incision. The retrorectus plane between the rectus muscle and posterior rectus sheath is developed laterally using electrocautery until the perforating neurovascular bundles are encountered just medial to the linea semilunaris. Care must be taken to avoid injury to the bowel during this dissection if the stoma was left in situ. The posterior lamella of the internal abdominal oblique aponeurosis is incised just medial to the neurovascular bundles to expose the transversus abdominis muscle. The transversus abdominis may be divided cephalad to caudad or vice versa, but it is important to recognize that it transitions from muscular fibers in the upper abdomen to aponeurotic fibers in the mid-abdomen and does not contribute to the posterior rectus sheath below the arcuate line. Once the transversus abdominis is divided along its length, the preperitoneal or pretransversalis plane can be developed laterally to the psoas, superiorly to the costal margin and central tendon of the diaphragm, and inferiorly to the retropubic space using sharp or blunt dissection of the transversus abdominis muscle fibers from the peritoneum below. The goals of this dissection are to reduce tension on the posterior rectus sheath closure and to allow for an adequate pocket for the mesh. The area behind the stoma is most easily addressed by dividing the transversus abdominis superior and inferior to the aperture, and then merging those planes laterally.

### Mesh placement

Commonly used mesh configurations include the keyhole, cruciate, and retromuscular Sugarbaker techniques. We typically use a mediumweight polypropylene mesh that is at least 30 cm × 30 cm placed in a diamond configuration to allow for at least 5 cm of hernia overlap in all directions. Our practice is to preferentially use a retromuscular Sugarbaker mesh configuration unless there is inadequate bowel and/or mesenteric length or in the case of a continent urostomy, in which case a keyhole or cruciate mesh configuration is used.

### Keyhole configuration

**Fig. 1:**
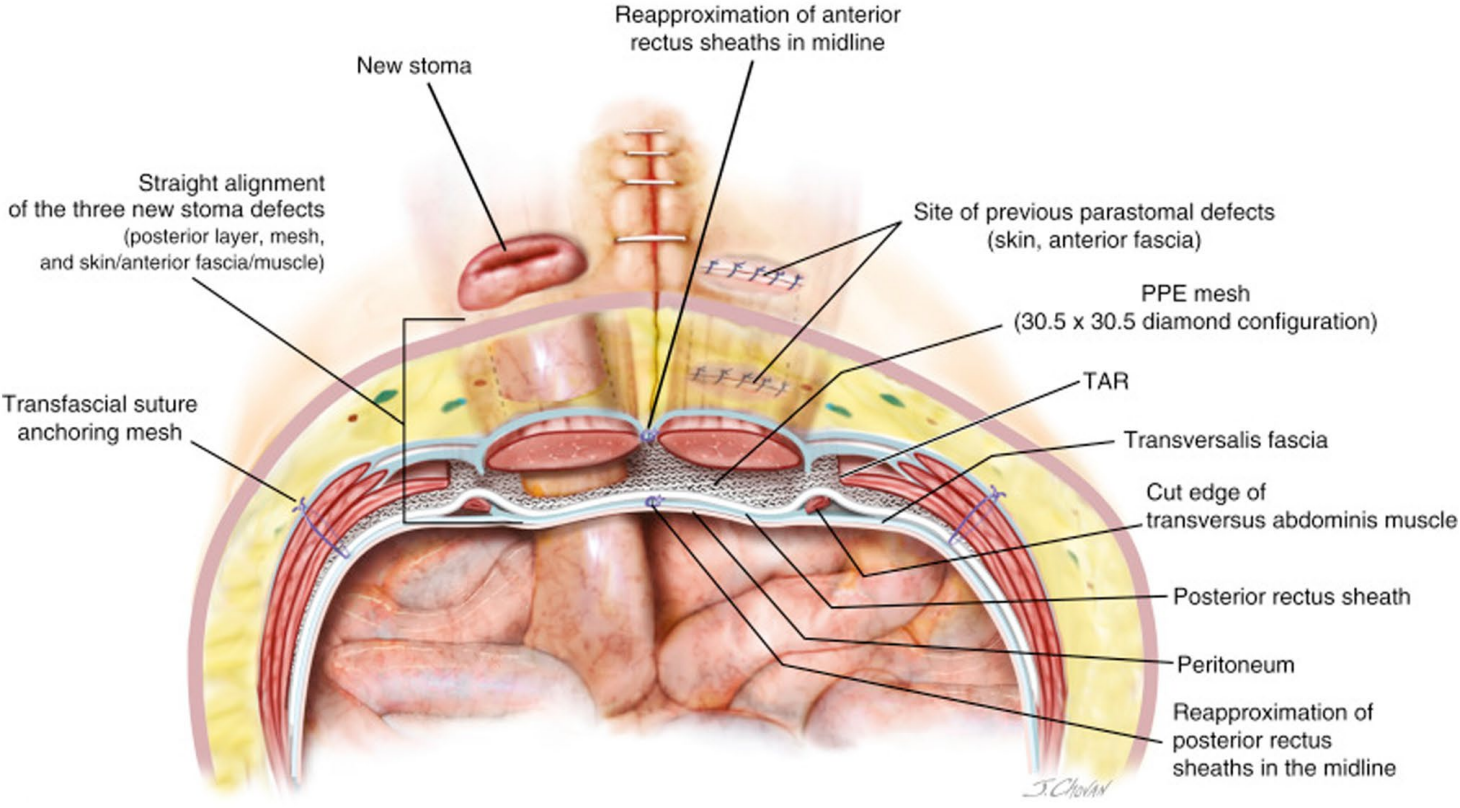
Cruciate mesh configuration (Annotation: transfascial fixation is demonstrated here but is not routinely performed in our practice)

For stomas left in situ, a keyhole mesh configuration may be used (Fig. 1). The stoma aperture in the posterior sheath is tightened just enough to accommodate the stoma bowel and then the posterior rectus sheath is closed using a running absorbable suture. The anterior fascial defect is tightened with the figure of eight slowly absorbable sutures. We then place the mesh in the retromuscular space and a slit is made in it to accommodate the bowel. Care is taken at this step to ensure that the aperture in the mesh is aligned with the anterior and posterior sheath apertures to prevent “scissoring’ of the bowel. The free ends of the slit may be closed behind the stoma bowel using a permanent suture.

### Cruciate configuration

For stomas that are taken down and re-matured, a cruciate mesh configuration may be used. The stoma bowel is brought through a fenestration in the peritoneum and the posterior rectus sheath is closed. A cruciate incision is then made in the mesh where the bowel is anticipated to pass through it. The bowel is pulled through the cruciate incision and the mesh is placed in the retromuscular space. The bowel is then gently pulled through the anterior aperture. All three apertures—the posterior fascia, mesh, and anterior fascia—must be aligned to prevent mesh erosion into the stoma. The apertures should also be just large enough to accommodate the bowel.

### Retromuscular sugarbaker

**Fig. 2:**
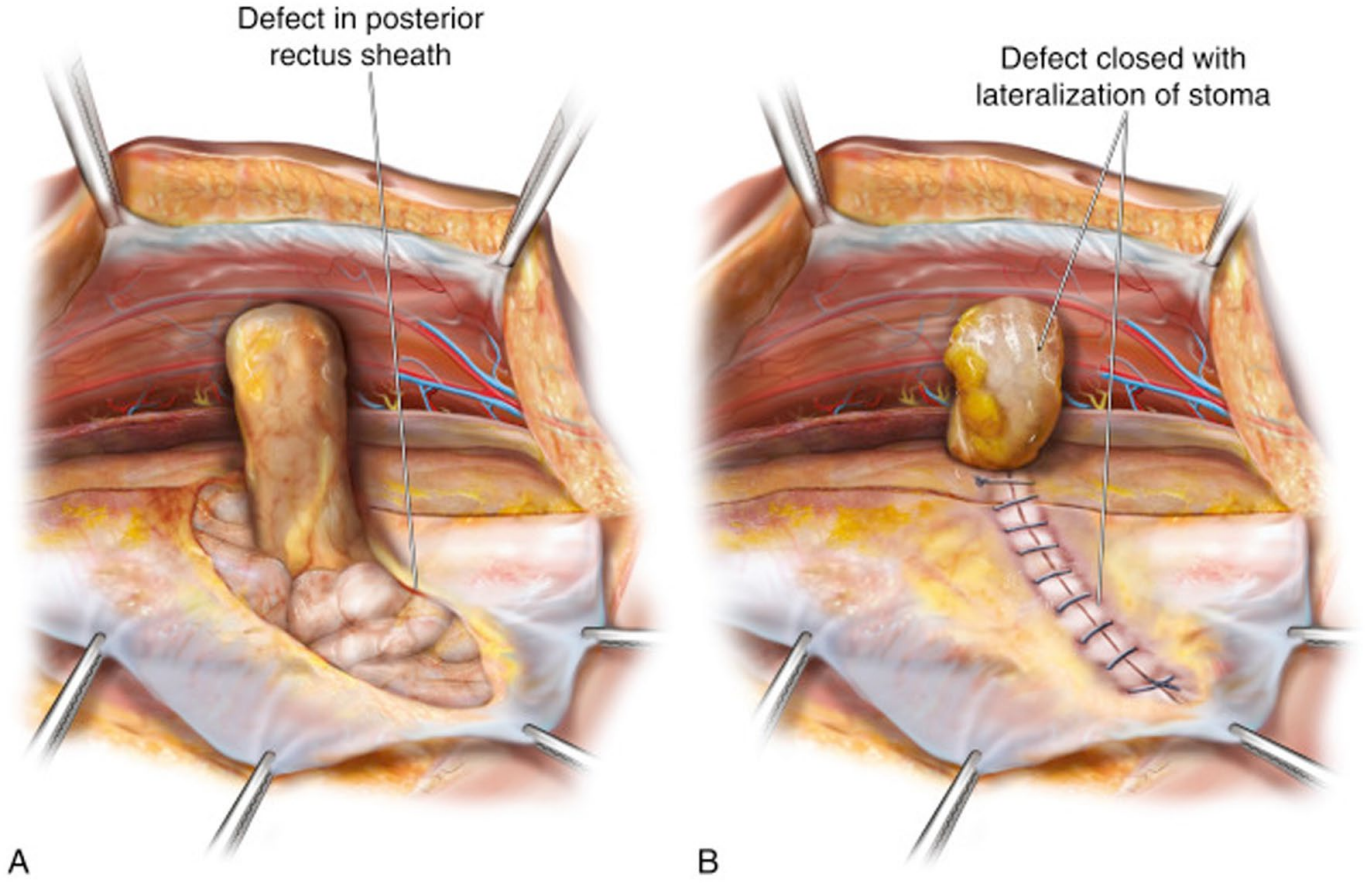
Lateralization of the stoma

**Fig. 3:**
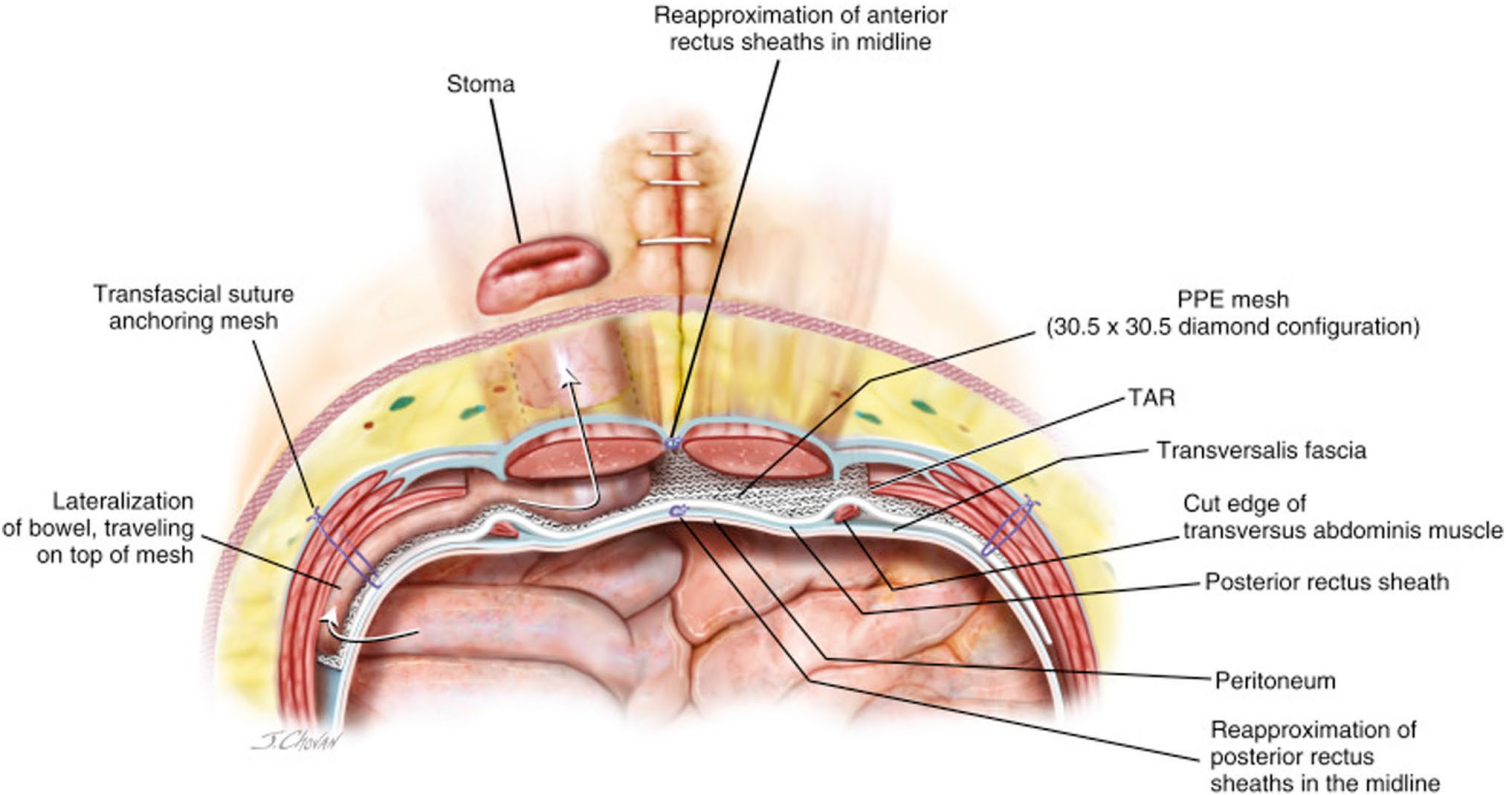
Sugarbaker mesh configuration (Annotation: transfascial fixation is demonstrated here but is not routinely performed in our practice)

Before selecting a retromuscular Sugarbaker mesh configuration, we verify that there is adequate bowel length to allow for lateralization. If the stoma is left in situ, the peritoneum is slit laterally, the stomal bowel is moved laterally in the retromuscular space, and then the peritoneum is closed medially to the bowel with running absorbable suture (Fig. 2). This allows the posterior fascial aperture to be offset from the anterior fascial aperture. The anterior fascial defect is tightened with a figure of eight slowly absorbable sutures. If the stoma is taken down, then a new lateral opening can be created in the peritoneum to pull the bowel through prior to closing the posterior rectus sheath. The mesh is placed in the retromuscular space in, often in a diamond configuration (Fig. 3). The mesh can be allowed to abut the stoma at the lateral edge or it can be slit to wrap around the stoma. If the mesh is slit, we do not sew the free ends together behind the stoma bowel.

We generally avoid fixating the mesh near the stoma bowel given our previous experiences with mesh erosions, which were attributed to transfascial fixation sutures [8].

### Closure

If the stoma is left in situ, the defect is tightened around the bowel using large, slowly absorbable sutures in a figure-of-eight pattern. We typically leave two closed suction drains in the retromuscular space and subcutaneous drains are placed at the discretion of the surgeon. The linea alba is approximated using large, slowly absorbable sutures in a series of interrupted figure-of-eights. The soft tissue is closed in layers with absorbable sutures. In cases of stoma relocation, the fascial defects from old stoma sites are loosely closed in a purse string fashion. Finally, the stoma is matured.

### Postoperative care

Postoperatively, patients are admitted to a surgical floor and placed on an enhanced recovery pathway [9]. Patients are started on clear liquids on the day of surgery and progressed as tolerated. Multimodal pain control is implemented with a goal to minimize opioid analgesia. Antibiotic management is at the discretion of the surgeon but is generally not continued beyond 24 h postoperatively. Retromuscular drains are typically removed when the output is 50 mL/day or prior to discharge.

## Results

Our institution has previously published our outcomes following parastomal hernia repairs. Our early institutional experiences with both keyhole and retromuscular Sugarbaker repairs showed promising recurrence rates of 11% at 13 months, but significant wound morbidity and stoma-related complications of necrosis, bowel obstruction, and perforation after both techniques [8, 10]. Early outcomes of our randomized controlled trial of keyhole and Sugarbaker mesh configurations for open retromuscular parastomal hernia repairs found a 16% rate of wound complications requiring procedural intervention and a 6.7% reoperation rate within 90 days postoperatively. Stoma-related complications were observed, including necrosis, distal obstruction, and mucocutaneous separation. Although the morbidity was high for all patients, there were no significant differences between keyhole and Sugarbaker techniques [5]. A post hoc analysis of another randomized controlled trial at our institution suggested a recurrence advantage for the Sugarbaker technique over keyhole (11% vs 30%) recurrence at 2 years, respectively [11]. This possible recurrence advantage is currently under investigation in a randomized controlled trial [12].

## Discussion

Here, we describe our approach to complex parastomal hernias. There are multiple advantages to the open retromuscular approach. Retromuscular placement of mesh, as compared to alternative mesh locations, is likely associated with recurrence and wound complication advantages [13]. Patients with parastomal hernias in the setting of permanent stomas often have concomitant midline defects and hernias from previous stoma sites, which may all be addressed through an open retromuscular approach.

There are a few key considerations when performing these repairs. One consideration is to avoid twisting of the stoma bowel which may compromise the blood supply, cause mechanical obstruction, or disrupt the mucocutaneous junction. Additionally, whether performing a keyhole or retromuscular Sugarbaker repair, the apertures in the fascial layers should be snug enough to just allow the stoma bowel passage without additional bowel loops. Care should be taken in the placement of the mesh to avoid excessive tension or misalignment between the stoma bowel and the mesh that may lead to mesh erosion. There is a lack of evidence in the literature regarding optimal stoma disposition management, so at this time the decision to leave the stoma in situ versus rematuring or resiting the stoma is left to the discretion of the surgeon and the preference of the patient.

We advocate that surgeons attempting these repairs should have advanced training in abdominal wall surgery. Component separations for ventral hernias require a thorough understanding of abdominal wall anatomy and familiarity with operating in multiple reoperative tissue planes. The presence of a stoma increases the technical difficulty of components separation. Multidisciplinary collaboration with colorectal and urological surgeons is important for identifying additional interventions that may be needed and for optimizing the timing of repair. We also want to emphasize the importance of engaging perioperative care teams, including anesthesia staff, nurses, intensive care units, and stoma nurses to optimize diagnosis and management of complications.

We are not advocating for this open, retromuscular approach in all patients with parastomal hernias. Retromuscular repairs have many advantages but should be utilized judiciously as recurrence rates may approach 20% and redo-retromuscular hernia repairs are associated with significant morbidity [14]. Considering the high incidence and persistently high recurrence rates for parastomal hernias, it is important to carefully assess patients and their symptoms prior to recommending repair. Laparoscopic and robotic repairs have been described and are alternative approaches that may be considered for patients with small to medium parastomal defects [15, 16]. While surgeons within our institution have experience with laparoscopy and robotic surgery, the population presenting to our center tends to be complex and less amenable to minimally invasive approaches. In the setting of acute incarceration or mesh infection, there are often multiple barriers to successful optimization for reconstruction, including limited time to fully evaluate the patient, increased contamination, and in some cases medical instability. In these cases, we favor a primary parastomal repair, with definitive abdominal wall reconstruction delayed until the patient is optimized [6].

## Summary

Parastomal hernias can be challenging for even experienced abdominal wall surgeons. While more prospective trials are needed to improve outcomes and identify ideal approaches, surgeons can optimize results through comprehensive preoperative patient evaluation and planning, meticulous intraoperative technique, and careful postoperative management.
